# Automated spectroscopic modelling with optimised convolutional neural networks

**DOI:** 10.1038/s41598-020-80486-9

**Published:** 2021-01-08

**Authors:** Zefang Shen, R. A. Viscarra Rossel

**Affiliations:** Soil and Landscape Science, School of Molecular and Life Sciences, Curtin University, GPO Box U1987, Perth, WA 6845 Australia

**Keywords:** Agroecology, Environmental impact, Carbon cycle

## Abstract

Convolutional neural networks (CNN) for spectroscopic modelling are currently tuned manually, and the effects of their hyperparameters are not analysed. These can result in sub-optimal models. Here, we propose an approach to tune one-dimensional CNN (1D-CNNs) automatically. It consists of a parametric representation of 1D-CNNs and an optimisation of hyperparameters to maximise a model’s performance. We used a large European soil spectroscopic database to demonstrate our approach for estimating soil organic carbon (SOC) contents. To assess the optimisation, we compared it to random search, and to understand the effects of the hyperparameters, we calculated their importance using functional Analysis of Variance. Compared to random search, the optimisation produced better final results and showed faster convergence. The optimal model produced the most accurate estimates of SOC with $\hbox {RMSE} = 9.67 \pm 0.51$ (s.d.) and ${R}^2 = 0.89 \pm 0.013$ (s.d.). The hyperparameters associated with model training and architecture critically affected the model’s performance, while those related to the spectral preprocessing had little effect. The optimisation searched through a complex hyperparameter space and returned an optimal 1D-CNN. Our approach simplified the development of 1D-CNNs for spectroscopic modelling by automatically selecting hyperparameters and preprocessing methods. Hyperparameter importance analysis shed light on the tuning process and increased the model’s reliability.

## Introduction

Soil is a complex matrix of varying organic and mineral composition, water and with different particle sizes. Soil spectra in the visible and infrared can be used to characterise soil. These spectra result from the interactions between electromagnetic radiation (between 400 to ~20,000 nm) and soil chemical and physical components^1^. Since a spectrum contains information on the fundamental compositing of soil, spectra can be used to estimate functional soil properties such as soil organic carbon (SOC). Diffuse reflectance in the visible and near infrared (vis–NIR) has been used for this purpose. The technique is rapid, cost-effective, non-destructive, and non-polluting. Additionally, a single spectrum, can be used to estimate different soil properties including SOC, soil texture (clay, sand and silt contents), water, cation exchange capacity, and pH^2^. To estimate soil properties with spectra, one needs to develop spectroscopic models using a spectral library. For this reason, many soil spectral libraries have been developed, at various scales, i.e. global^3^, continental^4^, national^5–10^ and regional^11^.

Vis–NIR spectra are highly collinear and absorptions are broad and overlapping, making it difficult to assign specific spectral features to soil properties^2^. Various spectral preprocessing methods are commonly used to remove noise, extract information from the spectra and improve the performance of the spectroscopic models, which describe the establishment of the relationship between the spectra and soil properties^2^. Statistical methods, such as partial least squares regression^12^, have been used to model soil properties with spectra. More recently, other non-linear methods including machine learning have also been used^13^. They include methods such as random forest^14^, support vector machine^15^, artificial neural networks^16^.

Recent spectroscopic modelling has shown that convolutional neural networks (CNNs) can potentially outperform conventional statistical and machine learning models. CNNs are a subset of deep learning, with successful applications in domains such as image classification^17^, video analysis^18^, drug discovery^19^. They are well-known for extracting useful local correlations in the data^20^. To utilise this feature for soil property prediction with spectra, researchers have developed various CNNs with one-dimensional (1D-CNNs)^21–23^ and two-dimensional structures (2D-CNNs)^24–26^. The 1D-CNNs process one-dimensional arrays, e.g. soil spectra, whereas 2D-CNNs use two-dimensional data with contextual information. Spectrograms derived from soil spectra were used in 2D-CNNs^24^. However, these spectrograms, which are essentially an image of stacked spectra, lack that 2D contextual information used in 2D-CNNs. So, unsurprisingly, 1D-CNNs produced more accurate spectroscopic predictions of soil properties compared to 2D-CNNs^23^.

A typical CNN can have a large number of hyperparameters, and their selection is critical to the CNN’s performance. To find the set of hyperparameters that generates optimal model performance, one needs to employ hyperparameter tuning, or optimisation^27^ (HPO). None of the CNNs for soil spectroscopic modelling reported in the literature^21–24,26^ have undertaken a thorough hyperparameter search, often relying on only manual tuning. Not using HPO may fail to fully exploit the capability of CNNs, and the lack of information about the tuning process can also affect the reliability of the models. Additionally, the manual tuning used in these studies can be tedious and time-consuming.

Here, we propose a framework to automatically optimise the tuning of hyperparameters in 1D-CNNs and to obtain insights into the tuning process. The aims of our study are to: (i) establish a parametric representation of 1D-CNNs for modelling SOC with a large spectral library, (ii) use a Bayesian optimisation algorithm to derive an optimal 1D-CNN architecture, maximise model performance and accurate estimates of SOC, and (iii) conduct hyperparameter importance analysis to better understand the effect of various hyperparameters on the model’s performance.

## Hyperparameter optimisation of 1D-CNNs

### 1D-CNN hyperparameters

A typical 1D-CNN consists of a number of convolutional, pooling, and fully-connected layers. Batch normalisation^28,29^ and Dropout^30^ layers can also be included for faster training and better generalisation. A 1D-CNN is defined by specifying the relative locations of these layers and their associated hyperparameters.

A convolutional layer has hyperparameters that describe the number of filters, the kernel size, the stride, and the type of padding to use. The number of filters determines the number of feature maps that can be generated after a convolution; the kernel size gives the size of the filters; the stride defines the step size for sliding the kernel along the input array; the padding type specifies the method for dealing with the input arrays’ borders (e.g. by adding zeros).

A pooling layer has hyperparameters that are similar to those in a convolutional layer. In this case, they describe the pooling type, that is, Average pooling or Max pooling, the pool size to define the size of the pooling region, the stride size and the type of padding to use. A fully-connected layer has one hyperparameter, that is, the number of units in the layer. A batch normalisation layer has no hyperparameters. A dropout layer has one hyperparameter, the dropout rate, which is the probability to retain a unit.

Nonlinear activations enable neural networks to learn complex relationships between features (or predictors) and targets (or predictands). Commonly used activations are ReLU^31,32^, Leaky Rectified Linear Unit (LeakyReLU)^33^, exponential linear unit (ELU)^34^, scaled exponential linear unit (SELU)^35^, and Swish^36^. Modern deep neural networks use mini-batch gradient descent to update their weights^37–40^. Several optimisers have been developed for this purpose, such as Adagrad^41^, RMSprop^37^, and Adam^42^. Batch size is the number examples in a mini-batch. There usually exists a threshold after which the model starts to deteriorate^43^.

### Hyperparameter optimisation (HPO)

HPO aims to solve the problem of finding the best set of hyperparameters that maximise a model’s performance^27^. It can be expressed as:

1 $$\begin{aligned} \varvec{\lambda }^*=arg \min _{\varvec{\lambda } \in \varvec{\Lambda }} \frac{1}{k} \sum _{i=1}^{k} \varvec{L}\left( \varvec{\lambda }, \varvec{D}_{train}^{(i)}, \varvec{D}_{valid}^{(i)}\right) \end{aligned}$$

where $\varvec{\lambda }$ denotes a vector of hyperparameters; $\varvec{\lambda }^*$, the vector of the optimal hyperparameters; $\varvec{\Lambda }$, the hyperparameter space; *k*, the number of folds used in cross-validation; $\varvec{L}$, the objective function for evaluating model performance on each fold; $\varvec{D}_{train}^{(i)}$ and $\varvec{D}_{valid}^{(i)}$, the datasets for training and validation in the* i*th fold, respectively.

Current HPO methods include manual search, grid search^44,45^, random search^46^, gradient-based methods^47–51^, Bayesian optimisation^52–54^, adaptive resource allocation^55,56^, and population-based methods^57,58^. The most important criterion for choosing a HPO algorithm is the nature of the hyperparameter space. The hyperparameter space in spectroscopic modelling is high-dimensional, mixed-typed (continuous and categorical hyperparameters), and tree-structured (conditional hyperparameters). It is an extremely complex space that many algorithms fail to work on. Sequential Model-based Algorithm Configuration (SMAC) and Tree Parzen Estimators (TPE) are able to search through such a hyperparameter space efficiently^59^. The TPE algorithm is also suitable for hyperparameter importance analysis as it evaluates all the folds in cross-validation and returns the overall objective for each hyperparameter configuration.

#### Bayesian optimisation—the TPE algorithm

A Bayesian optimisation algorithm consists of two main components: a surrogate function and a performance metric. The surrogate function is a computationally efficient approximation of the real objective function, which updates as the optimisation progresses. The performance metric, together with the surrogate function, proposes a new hyperparameter configuration for the next evaluation. The TPE algorithm uses Tree Parzen Estimator as the surrogate function and the Expected Improvement as the performance metric. Details about the TPE algorithm can be found in^52^.

The TPE algorithm starts with a number of evaluations on randomly sampled hyperparameter configurations. The optimisation history ($\varvec{H}$), which consists of hyperparameter vector and objective value pairs, can then be established using the completed evaluations. The algorithm then uses $\varvec{H}$ to update the surrogate function and to select the next configuration of the hyperparameters ($\varvec{\lambda _N}$). After the evaluation of $\varvec{\lambda _N}$, the optimisation history $\varvec{H}$ is updated. The algorithm continues proposing and evaluating new hyperparameter configurations until a stopping criterion is reached. The TPE algorithm is summarised in Algorithm 1.

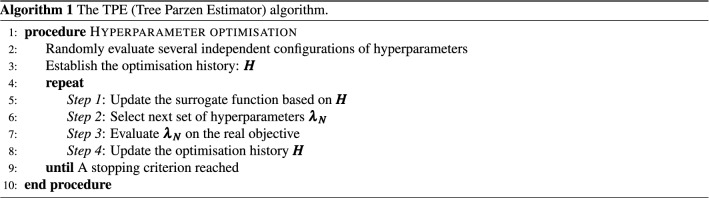



## Results

In the Methods section below, we describe the dataset used, the parametric representation of the 1D-CNN, the hyperparameters, their values, and the optimisation.

### Hyperparameter search histories

**Figure 1:**
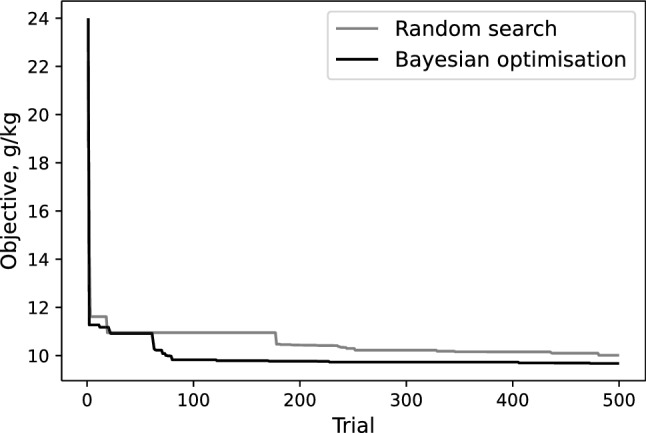
Comparison of the hyperparameter search history from random search and the Bayesian optimisation.

Hyperparameter search histories for random search and Bayesian optimisation are compared in Fig. 1. Both the objective values in the search methods decreased as hyperparameter search progressed. The Bayesian optimisation plateaued at 80 trials (9 h and 51 min) whereas random search needed 250 trials to level-off (24 h and 12 min). The Bayesian optimisation approach was approximately 3.1 and 2.5 times faster than random search in terms of number of trials and computational time, respectively. It returned a smaller minimum objective than random search (Fig. 1).

### Optimal hyperparameters

The hyperparameters of the optimal model are given in Table 1. Based on the input channel states, the model took four input channels: Reflectance, Absorbance, Absorbance + First derivative (ABS + D1), and Absorbance + Second derivative (ABS + D2) (see Methods, Fig. 7). The model involved only one convolutional block (Conv-block). It is simply a convolutional layer with an activation function. Batch normalisation, pooling, and dropout layers were all disabled in the Conv-block. The model used two Fully-connected blocks (FC-blocks) and batch normalisation and dropout layers were turned on in the FC-blocks.

**Table 1:** Optimal 1D-CNN hyperparameters.

Group	Hyperparameter	Optimal value	Value range
Input channel	Reflectance state	ON	[ON, OFF]
	Absorbance state	ON	[ON, OFF]
	ABS + D1 state	ON	[ON, OFF]
	ABS + D2 state	ON	[ON, OFF]
	ABS + SNV state	OFF	[ON, OFF]
Conv-block	Filters	55	[4, 64]
	Kernel size	5	[2, 10]
	Stride size	4	[2, 10]
	Conv padding type	Same	[Same, Valid]
	Batch normalisation state	OFF	[ON, OFF]
	Activation	SELU	[ReLU, LeakyReLU, ELU, SELU, Swish]
	Pooling state	OFF	[ON, OFF]
	Dropout state	OFF	[ON, OFF]
FC-block 1	Number of nodes	251	[5 , 256]
	Batch normalisation state	ON	[ON, OFF]
	Activation	ELU	[ReLU, LeakyReLU, ELU, SELU, Swish]
	Dropout state	ON	[ON, OFF]
FC-block 2	Number of nodes	219	[5, 256]
	Batch normalisation state	ON	[ON, OFF]
	Activation	Swish	[ReLU, LeakyReLU, ELU, SELU, Swish]
	Dropout state	ON	[ON, OFF]
Others	FC-block dropout rate	0.22	[0.0, 0.5]
	Optimiser	Adam	[Adagrad, RMSProp, Adam]
	Batch size	1105	[16, 17607]
	Epochs	1448	[100, 1500]

### Architecture and performance

**Figure 2:**
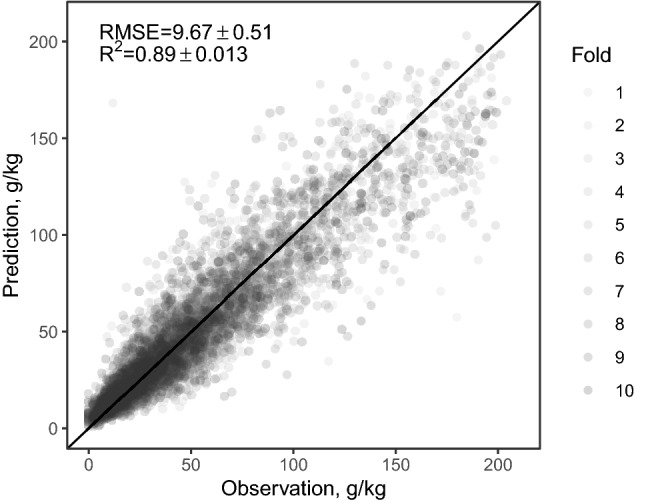
Observed vs predicted soil organic carbon (SOC) from a 10-fold cross-validation. The solid 1:1 line is drawn for reference.

The architecture of the optimal 1D-CNN is shown in Table 2. Compared to other published 1D-CNNs^21,23^, our optimised model is very simple. Interestingly, such a simple model achieved the best performance in our study. The cross-validated SOC predictions are shown in Fig. 2. The optimal model produced an RMSE of $9.67 \pm 0.51$ (s.d.) and an ${R}^2$ of $0.89 \pm 0.013$ (s.d.).

**Table 2:** Optimal 1D-CNN architecture.

Layer type	Kernel size	Filters	Padding type	Strides	Output width	Activation
Convolutional	(5, 1)	55	Same	4	53	SeLU
Flatten	–	–	–	–	2915	–
Dense	–	–	–	–	251	ELU
Batch normalisation	–	–	–	–	–	–
Dropout	–	–	–	–	–	–
Dense	–	–	–	–	219	Swish
Batch normalisation	–	–	–	–	–	–
Dropout	–	–	–	–	–	–
Dense	–	–	–	–	1	Linear

### Hyperparameter importance

**Figure 3:**
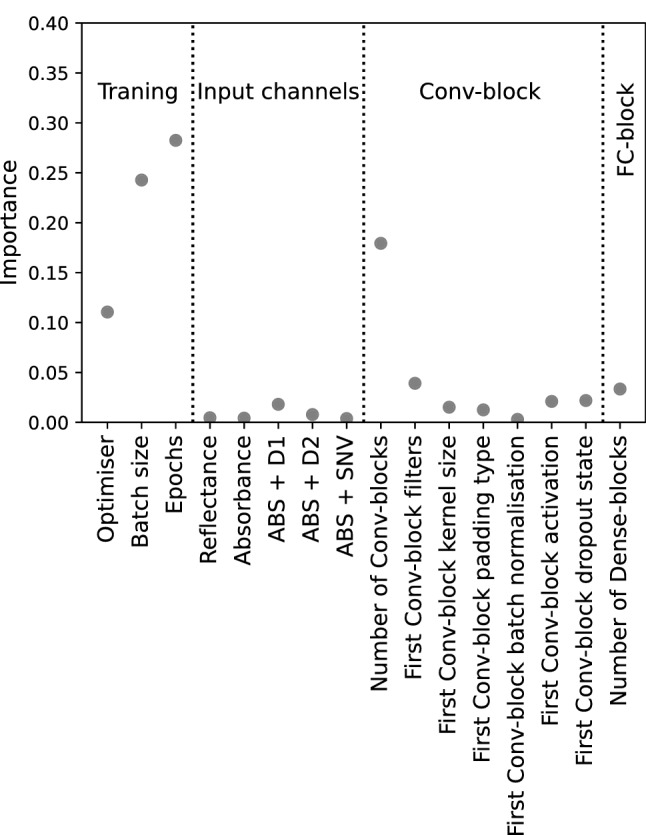
Hyperparameter importance based on the optimisation history. Hyperparameters are classified into training-related, input channels, Conv-block, and FC-block.

Importance of the hyperparameters are presented in Fig. 3. Training-related hyperparameters (optimiser, batch size, and number of epochs) greatly affected the performance of the model. Model architecture was also critical to model performance, shown by the larger relative importance of Conv-block numbers (Fig. 3). Other architectural hyperparameters had a lesser impact on the model. In terms of preprocessing methods, only ABS + D1 had slight importance while the importance of other methods were negligible. Thus, only a few hyperparameters were critical to the model’s performance, which aligns with previous findings^46,60^.

**Figure 4:**
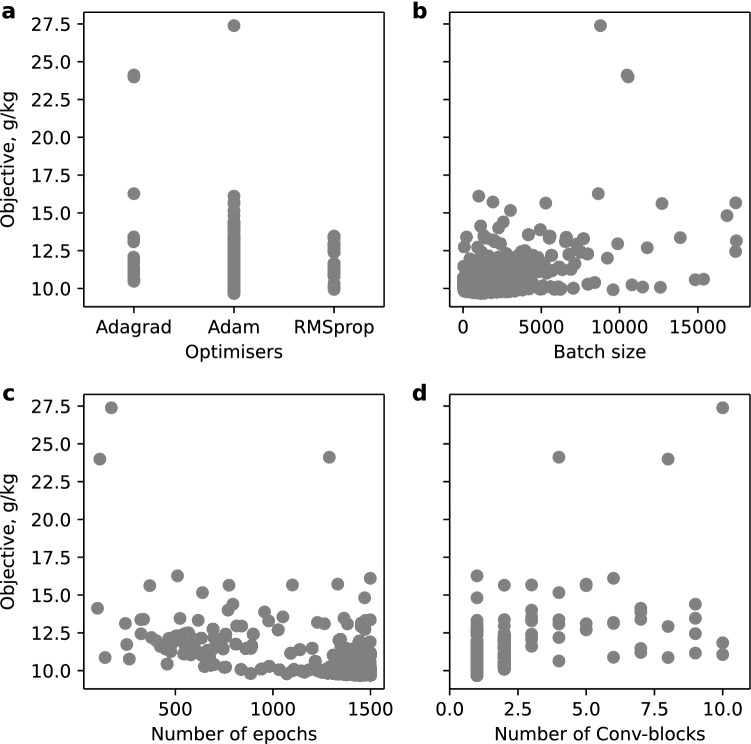
Trials on the important hyperparameters. (**a**) Optimisers. (**b**) Batch size. (**c**) Number of epochs. (**d**) Number of Conv-blocks.

We further investigated the hyperparameters’ effects by visualising the trials on the important hyperparameters, as in Fig. 4. Each dot represents a trial given by certain hyperparameters. The optimisation preferred optimiser Adam, batch size around 1000, number of epochs close to 1500, and only one Conv-block, which aligns with the optimal hyperparameters in Table 1. The optimisation covered the entire hyperparameter value ranges suggesting a thorough search. It is also obvious that more evaluations happened around the optimal values, which indicates that TPE algorithm can focus on promising values rather than testing values randomly.

## Discussion

The parametric representation allowed us to construct 1D-CNNs based on a given set of hyperparameters. It enabled the establishment of an optimisation problem that can automatically discover the best hyperparameters. The TPE algorithm could search through a high-dimensional, mixed-typed, and tree-structured hyperparameter space. It converged quickly and achieved better results compared to random search. The method is also practical in terms of computation time. The parametric representation and Bayesian optimisation combined, simplified the development of 1D-CNNs for the spectroscopic modelling by automating the process. The framework is also suitable for other fixed-length 1D signals in different soil and environmental sciences and engineering applications.

Hyperparameter importance analysis can shed light on the model tuning process, which is often assumed to be “black-box”. It calculates the importance of the individual hyperparameters and identifies the most important ones. This informed hyperparameter search can increase our understanding of the trained model and its reliability.

The optimised 1D-CNN produced accurate predictions of SOC. It delivered better estimates compared to other published studies that used the same LUCAS dataset^23,24^ (Table 3).

**Table 3:** Comparison of CNNs.

Model	Dataset	Validation	RMSE ($\hbox {g} \cdot \hbox {kg}^{-1}$)	${R}^2$
2D-CNN^24^	Organic + mineral samples	Test set	32.14	0.88
1D-CNN^23^	Organic samples	Cross-validation	11.31	0.85
This study	Organic + mineral samples	Cross-validation	$9.67 \pm 0.51$	$0.89 \pm 0.013$

The 2D-CNN and our optimised 1D-CNN were developed using the organic and mineral samples in the LUCAS dataset. The 1D-CNN^23^ was developed using only the organic samples.

Based on the hyperparameter importance of the input channels, preprocessing vis–NIR spectra with ABS + D1 can help to improve prediction accuracy. However, the influence of other preprocessing methods was insignificant, which implies that an optimised 1D-CNN itself can extract such information from the raw spectra. Therefore, preprocessing vis–NIR spectra with ABS + D1 may help the development of spectroscopic models using 1D-CNNs.

The simplicity of the optimal 1D-CNN suggests that spectra are composed of relatively simple and low-level features. This is consistent with the nature of vis–NIR spectra, which are characterised by broad and weak vibrational modes in the NIR and electronic transitions in the visible range, giving soil spectra few, broad absorption features. Attempting to extract complex and high-level features using multiple convolutional layers is likely to affect performance, as shown in Fig. 4d. The optimal 1D-CNN did not use a pooling layer. A pooling layer usually serves to down-sample input features and to introduce local translation invariance^20^. The down-sampling can be alternatively achieved by using bigger stride size in convolutional layers. Introducing translation invariance in soil spectroscopic modelling may affect model performance, as the features in vis–NIR spectrum are located at specific wavelengths.

There are an increasing number of studies comparing new machine learning models for soil spectroscopic modelling. Some claim that their specific architectures outperform others. However, it is difficult to determine whether a model is better purely because of its unique architecture, or because it might have been tuned better. The problem is greater when the models are tuned manually, since it is impossible to guarantee the same amount of tuning to all the models. Thus, assessment of such machine learning models could be biased. The optimisation method in this study provides a way for unbiased assessment of models, as it can fully exploit different architectures.

We presented an approach for optimal, automatic spectroscopy modelling with 1D-CNNs. It simplified the development of the 1D-CNNs. It searched thorough a complex hyperparameter space and returned an optimal 1D-CNN that produced accurate estimates of SOC. The hyperparameter importance analysis provided insights into the tuning process, which gave us confidence in the reliability of the model.

## Methods

### The dataset

**Figure 5:**
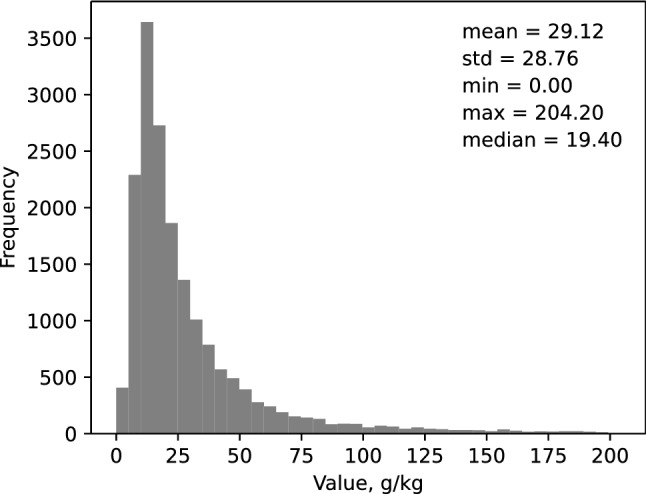
Histogram of the soil organic carbon (SOC) content in the LUCAS dataset ($\hbox {n} = 17{,}607$), showing moments of its distribution. The dataset includes both mineral and organic soil.

We used the Land Use/Land Cover Area Frame Survey (LUCAS) dataset which collected and analysed soil samples across Europe. Approximately 20,000 samples are publicly available each of which consists of a sample’s spectral signature and its corresponding physicochemical properties. The spectra cover a wavelength range from 400 to 2500 nm with resolution of 0.5 nm. We removed approximately 2000 samples from the downloaded LUCAS dataset because of errors that we could not explain in either the spectra or the soil properties or the geographic coordinates. Thus, the dataset that we used in our research has 17,607 samples. We downsampled the spectra by 1:20 to remove multicollinearily and to improve computational efficiency during the training of the models. Here, we only considered soil organic carbon (SOC) as the response, or target variable. The histogram of SOC’s distribution is shown in Fig. 5.

### A parametric representation for 1D-CNNs

**Figure 6:**
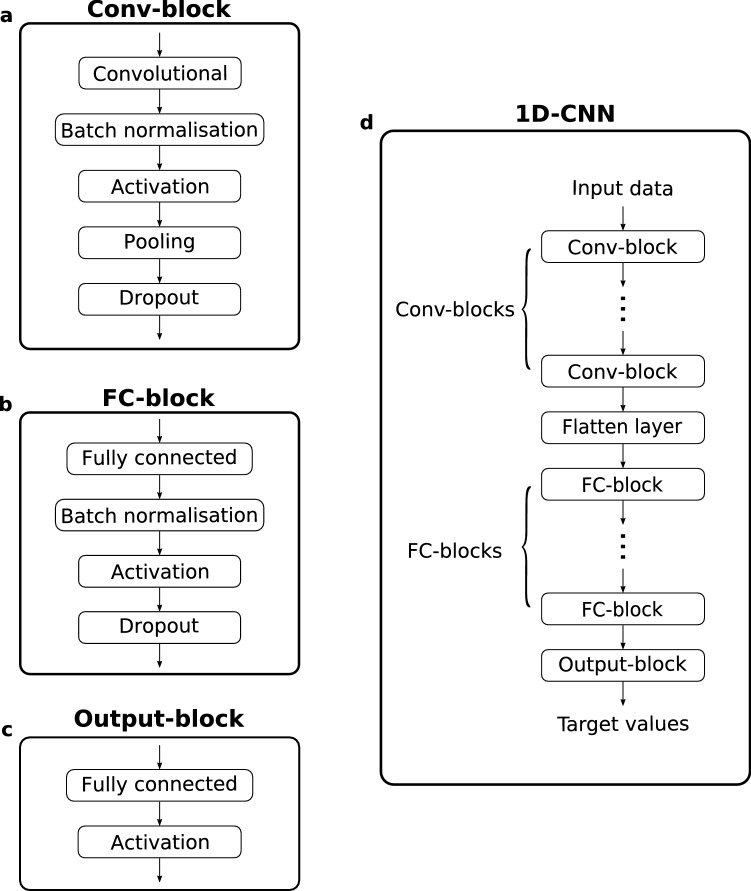
1D-CNN building blocks. (**a**) Convolutional block. (**b**) Fully-connected block. (**c**) Output block. (**d**) Construction of a 1D-CNN using the blocks.

We described 1D-CNNs using three types of building blocks: Convolutional blocks (Conv-blocks), Fully-connected blocks (FC-blocks), and an Output block (Fig. 6a–c). A Conv-block stacks a convolutional, batch normalisation, activation, pooling, and a dropout layer in sequence. Similarly, an FC-block consists of a fully-connected, a batch normalisation, an activation, and a dropout layer. The output block is essentially a Fully-connected layer that outputs target values. Thus, a 1D-CNN can be defined by a number of Conv-blocks and FC-blocks, joined by a Flatten layer (Fig. 6d).

**Figure 7:**
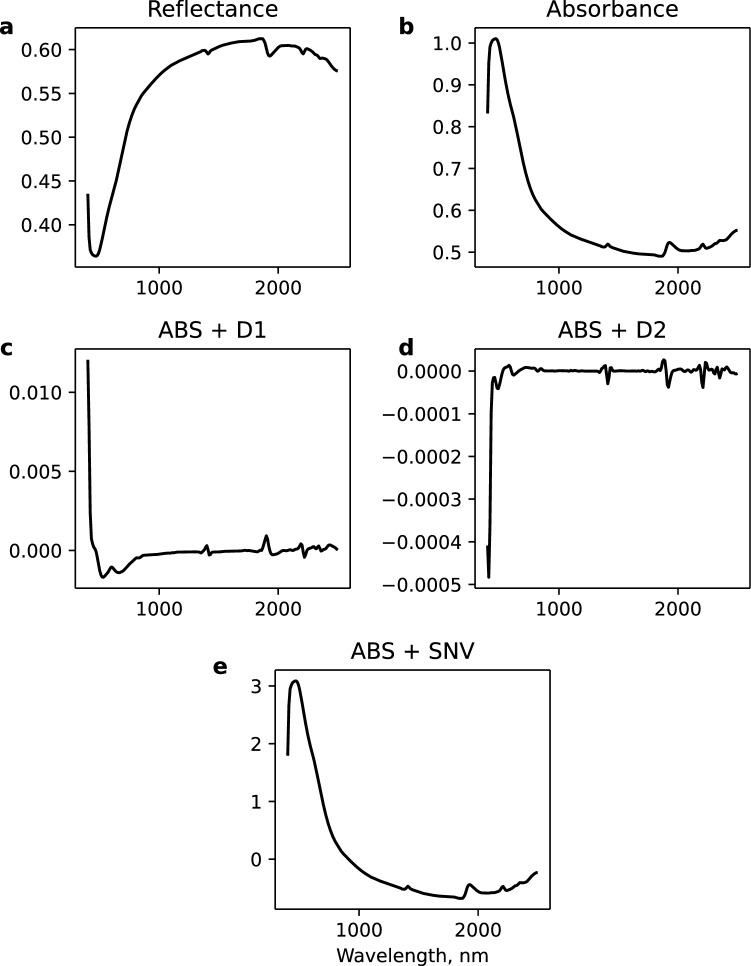
Input channels derived from different preprocessing methods. (**a**) Reflectance. (**b**) Absorbance. (**c**) Absorbance + first derivative. (**d**) Absorbance + second derivative (**e**) Absorbance + standard normal variate.

We used state hyperparameters to turn ON/OFF the Pooling, Batch Normalisation, and Dropout layers. To generate multiple input channels, we considered five common preprocessing methods: reflectance (R), absorbance ($= \hbox {log}~\hbox {R}^{-1}$), absorbance + first derivative (ABS+D1), absorbance + second derivatives (ABS+D2), and absorbance + standard normal variate (ABS+SNV). Each channel has a state hyperparameter (ON/OFF) to indicate whether to use the channel for the modelling. Figure 7 shows examples of the input channels.

The hyperparameters and their values are summarised in Table 4. The value ranges some hyperparameters can be predetermined, while others need to be determined specifically for the particular problem.

**Table 4:** 1D-CNN hyperparameters.

Group	Hyperparameter	Value range
Input channels	State	[ON, OFF]
Conv-block	Filters	–
	Kernel size	–
	Stride	–
	Conv padding type	[Same, Valid]
	Batch normalisation state	[ON, OFF]
	Activation	[ReLU, LeakyReLU, ELU, SELU, Swish, ...]
	Pooling state	[ON, OFF]
	Pool size	–
	Pooling padding type	[Same, Valid]
	Pooling strides	–
	Dropout state	[ON, OFF]
Flatten layer	–	–
FC-block	Number of nodes	–
	Batch normalisation state	[ON, OFF]
	Activation	[ReLU, LeakyReLU, ELU, SELU, Swish, ...]
	Dropout state	[ON, OFF]
Output layer	–	–
Others	Number of Conv-blocks	–
	Number of FC-blocks	–
	Conv-block Dropout rate	–
	FC-block Dropout rate	–
	Optimiser	[Adam, Adagrad, RMSProp, ...]
	Batch size	–
	Epochs	–

### Optimising 1D-CNNs for soil spectroscopic modelling

We used 10-fold cross-validation and the root mean squared error (RMSE) to fit and validate the 1D-CNNs for predicting SOC using the LUCAS dataset. From Eq. (1), the optimisation problem can then be expressed as:

2 $$\begin{aligned} \varvec{\lambda }^*=arg \min _{\varvec{\lambda } \in \varvec{\Lambda }} \frac{1}{10} \sum _{i=1}^{10} RMSE\left( \varvec{\lambda }, \varvec{D}_{train}^{(i)}, \varvec{D}_{valid}^{(i)}\right) \end{aligned}$$

where $\varvec{\Lambda }$ denotes the hyperparameter space; $\varvec{\lambda }$, a vector of hyperparameters in that space; $\varvec{\lambda }^*$, the vector of the optimal hyperparameters; $\varvec{D}_{train}^{(i)}$ and $\varvec{D}_{valid}^{(i)}$, the spectral datasets for train and validation in the* i*th split respectively. *RMSE* calculates the root mean squared error using the predicted and observed values in dataset $\varvec{D}_{valid}^{(i)}$, given a model fitted on $\varvec{\lambda }$ and $\varvec{D}_{train}^{(i)}$. The *RMSE* score on the 10 splits are averaged to form the overall objective.

Hyperparameters and their corresponding value ranges were those shown in Table 4. For the hyperparameters that are dataset-dependent, we set their value ranges based on recommended values in the literature and our experience with the dataset and 1D-CNNs. We employed Early Stopping^61^ when training the models to prevent overfitting. To assess the performance of the Bayesian optimisation-based approach we also ran a random search.

We conducted hyperparameter importance analysis to investigate the hyperparameters’ effects on the model performance. The hyperparameter importance was calculated using functional Analysis of Variance^60^, which requires an optimisation history that consists of records of hyperparameter configurations and its corresponding objective value. Since the algorithm computes the importance of the hyperparameters that exist in all records, we only calculated the importance for independent hyperparameters.

## References

[1] Ben-Dor E, Irons J, Epema G (1999). Soil reflectance. Remote Sens. Earth Sci. Man. Remote Sens..

[2] Soriano-Disla JM, Janik LJ, Viscarra Rossel RA, Macdonald LM, McLaughlin MJ (2014). The performance of visible, near-, and mid-infrared reflectance spectroscopy for prediction of soil physical, chemical, and biological properties. Appl. Spectrosc. Rev..

[3] Viscarra Rossel RA (2016). A global spectral library to characterize the world’s soil. Earth-Sci. Rev..

[4] Orgiazzi A, Ballabio C, Panagos P, Jones A, Fernández-Ugalde O (2018). Lucas soil, the largest expandable soil dataset for europe: a review. Eur. J. Soil Sci..

[5] Viscarra Rossel RA, Webster R (2012). Predicting soil properties from the australian soil visible-near infrared spectroscopic database. Eur. J. Soil Sci..

[6] Shi Z, Ji W, Viscarra Rossel RA, Chen S, Zhou Y (2015). Prediction of soil organic matter using a spatially constrained local partial least squares regression and the c hinese vis-nir spectral library. Eur. J. Soil Sci..

[7] Wijewardane NK, Ge Y, Wills S, Loecke T (2016). Prediction of soil carbon in the conterminous united states: visible and near infrared reflectance spectroscopy analysis of the rapid carbon assessment project. Soil Sci. Soc. Am. J..

[8] Peng Y (2013). Predicting soil organic carbon at field scale using a national soil spectral library. J. Near Infrared Spectrosc..

[9] Terra F. S., Demattê J. A., Viscarra Rossel R. A. (2015). Spectral libraries for quantitative analyses of tropical brazilian soils: comparing vis-nir and mid-ir reflectance data. Geoderma.

[10] Clairotte M (2016). National calibration of soil organic carbon concentration using diffuse infrared reflectance spectroscopy. Geoderma.

[11] Tziolas N, Tsakiridis N, Ben-Dor E, Theocharis J, Zalidis G (2019). A memory-based learning approach utilizing combined spectral sources and geographical proximity for improved vis-nir-swir soil properties estimation. Geoderma.

[12] Wold, Svante, Harold Martens, and Herman Wold. The multivariate calibration problem in chemistry solved by the pls method. *Matrix Pencils. Springer. *286–293 (1983).

[13] Viscarra Rossel RA, Behrens T (2010). Using data mining to model and interpret soil diffuse reflectance spectra. Geoderma.

[14] Lee S, Choi H, Cha K, Chung H (2013). Random forest as a potential multivariate method for near-infrared (nir) spectroscopic analysis of complex mixture samples: Gasoline and naphtha. Microchem. J..

[15] Devos O, Ruckebusch C, Durand A, Duponchel L, Huvenne J-P (2009). Support vector machines (svm) in near infrared (nir) spectroscopy: focus on parameters optimization and model interpretation. Chemom. Intell. Lab. Syst..

[16] Daniel K, Tripathi N, Honda K (2003). Artificial neural network analysis of laboratory and in situ spectra for the estimation of macronutrients in soils of lop buri (thailand). Soil Res..

[17] Rawat W, Wang Z (2017). Deep convolutional neural networks for image classification: a comprehensive review. Neural Comput..

[18] Ji S, Xu W, Yang M, Yu K (2012). 3d convolutional neural networks for human action recognition. IEEE Trans. Pattern Anal. Mach. Intell..

[19] Wallach, I., Dzamba, M. & Heifets, A. *Atomnet: A Deep Convolutional Neural Network for Bioactivity Prediction in Structure-Based Drug Discovery*. arXiv:1510.02855 (2015).

[20] LeCun Y, Bengio Y, Hinton G (2015). Deep learning. Nature.

[21] Veres, M., Lacey, G. & Taylor, G. W. Deep learning architectures for soil property prediction. In *2015 12th Conference on Computer and Robot Vision*, 8–15 (IEEE, 2015).

[22] Liu L, Ji M, Buchroithner M (2018). Transfer learning for soil spectroscopy based on convolutional neural networks and its application in soil clay content mapping using hyperspectral imagery. Sensors.

[23] Tsakiridis NL, Keramaris KD, Theocharis JB, Zalidis GC (2020). Simultaneous prediction of soil properties from vnir-swir spectra using a localized multi-channel 1-d convolutional neural network. Geoderma.

[24] Padarian J, Minasny B, McBratney A (2019). Using deep learning to predict soil properties from regional spectral data. Geoderma Reg..

[25] Padarian J, Minasny B, McBratney A (2019). Transfer learning to localise a continental soil vis-nir calibration model. Geoderma.

[26] Ng W (2019). Convolutional neural network for simultaneous prediction of several soil properties using visible/near-infrared, mid-infrared, and their combined spectra. Geoderma.

[27] Hutter F, Kotthoff L, Vanschoren J (2019). Automated Machine Learning: Methods, Systems, Challenges.

[28] Ioffe, S. & Szegedy, C. *Batch Normalization: Accelerating Deep Network Training by Reducing Internal Covariate Shift*. arXiv:1502.03167 (2015).

[29] Santurkar S, Tsipras D, Ilyas A, Madry A, Bengio S (2018). How does batch normalization help optimization?. Advances in Neural Information Processing Systems 31.

[30] Srivastava N, Hinton G, Krizhevsky A, Sutskever I, Salakhutdinov R (2014). Dropout: a simple way to prevent neural networks from overfitting. J. Mach. Learn. Res..

[31] Hahnloser RH, Sarpeshkar R, Mahowald MA, Douglas RJ, Seung HS (2000). Digital selection and analogue amplification coexist in a cortex-inspired silicon circuit. Nature.

[32] Nair, V. & Hinton, G. E. Rectified linear units improve restricted boltzmann machines. In *ICML* (2010).

[33] Maas, A. L., Hannun, A. Y. & Ng, A. Y. Rectifier nonlinearities improve neural network acoustic models. In *Proceedings of ICML* **30**, 3 (2013).

[34] Clevert, D.-A., Unterthiner, T. & Hochreiter, S. *Fast and Accurate Deep Network Learning by Exponential Linear Units (elus)*. arXiv:1511.07289 (2015).

[35] Klambauer G, Unterthiner T, Mayr A, Hochreiter S (2017). Self-normalizing neural networks. Adv. Neural Inf. Process. Syst..

[36] Ramachandran, P., Zoph, B. & Le, Q. V. *Searching for Activation Functions*. arXiv:1710.05941 (2017).

[37] Ruder, S. *An Overview of Gradient Descent Optimization Algorithms*. arXiv:1609.04747 (2016).

[38] Simonyan, K. & Zisserman, A. *Very Deep Convolutional Networks for Large-Scale Image Recognition*. arXiv:1409.1556 (2014).

[39] Pascanu, R., Mikolov, T. & Bengio, Y. On the difficulty of training recurrent neural networks. In *International Conference on Machine Learning* 1310–1318 (2013).

[40] Mnih, V. *et al.**Playing Atari with Deep Reinforcement Learning*. arXiv:1312.5602 (2013).

[41] Duchi, J., Hazan, E. & Singer, Y. Adaptive subgradient methods for online learning and stochastic optimization. *J. Mach. Learn. Res.* **12.7** (2011).

[42] Kingma, D. P. & Ba, J. *Adam: A Method for Stochastic Optimization*. arXiv:1412.6980 (2014).

[43] Keskar, N. S., Mudigere, D., Nocedal, J., Smelyanskiy, M. & Tang, P. T. P. *On Large-Batch Training for Deep Learning: Generalization Gap and Sharp Minima*. arXiv:1609.04836 (2016).

[44] Hsu, C.-W., Chang, C.-C., Lin, C.-J. *et al.* A practical guide to support vector classification. 1396–1400 (2003).

[45] Lerman P (1980). Fitting segmented regression models by grid search. J. R. Stat. Soc. Ser. C (Appl. Stat.).

[46] Bergstra J, Bengio Y (2012). Random search for hyper-parameter optimization. J. Mach. Learn. Res..

[47] Franceschi, L., Donini, M., Frasconi, P. & Pontil, M. *Forward and Reverse Gradient-Based Hyperparameter Optimization*. arXiv:1703.01785 (2017).

[48] Luketina, J., Berglund, M., Greff, K. & Raiko, T. Scalable gradient-based tuning of continuous regularization hyperparameters. In *International Conference on Machine Learning* 2952–2960 (2016).

[49] Maclaurin, D., Duvenaud, D. & Adams, R. Gradient-based hyperparameter optimization through reversible learning. In *International Conference on Machine Learning* 2113–2122 (2015).

[50] Bengio Y (2000). Gradient-based optimization of hyperparameters. Neural Comput..

[51] Domke, J. Generic methods for optimization-based modeling. In *Artificial Intelligence and Statistics* 318–326 (2012).

[52] Bergstra, J. S., Bardenet, R., Bengio, Y. & Kégl, B. Algorithms for hyper-parameter optimization. *Adv. Neural Inf. Process. Syst.* **24**, 2546–2554 (2011).

[53] Hutter, F., Hoos, H. H. & Leyton-Brown, K. Sequential model-based optimization for general algorithm configuration. In *International Conference on Learning and Intelligent Optimization*, 507–523 (Springer, 2011).

[54] Snoek J, Larochelle H, Adams RP (2012). Practical bayesian optimization of machine learning algorithms. Adv. Neural Inf. Process. Syst..

[55] Jamieson, K. & Talwalkar, A. Non-stochastic best arm identification and hyperparameter optimization. In *Artificial Intelligence and Statistics* 240–248 (2016).

[56] Li L, Jamieson K, DeSalvo G, Rostamizadeh A, Talwalkar A (2017). Hyperband: a novel bandit-based approach to hyperparameter optimization. J. Mach. Learn. Res..

[57] Loshchilov, I. & Hutter, F. *Cma-es for Hyperparameter Optimization of Deep Neural Networks*. arXiv:1604.07269 (2016).

[58] Jaderberg, M. *et al.**Population Based Training of Neural Networks*. arXiv:1711.09846 (2017).

[59] Eggensperger, K. *et al.* Towards an empirical foundation for assessing bayesian optimization of hyperparameters. In *NIPS Workshop on Bayesian Optimization in Theory and Practice* **10**, 3 (2013).

[60] Hutter, F., Hoos, H. & Leyton-Brown, K. An efficient approach for assessing hyperparameter importance. In *International Conference on Machine Learning* 754–762 (2014).

[61] Prechelt L (1998). Early Stopping-but When? In Neural Networks: Tricks of the Trade.

